# A prospective randomized clinical trial to assess antibiotic pocket irrigation on tissue expander breast reconstruction

**DOI:** 10.1128/spectrum.01430-23

**Published:** 2023-09-27

**Authors:** Jennifer N. Walker, Blake M. Hanson, Tayler Hunter, Shelby R. Simar, Jesus M. Duran Ramirez, Chloe L. P. Obernuefemann, Rajiv P. Parikh, Marissa M. Tenenbaum, Julie A. Margenthaler, Scott J. Hultgren, Terence M. Myckatyn

**Affiliations:** 1 Department of Microbiology and Molecular Genetics, McGovern Medical School, University of Texas Health Sciences Center, Houston, Texas, USA; 2 Department of Epidemiology, Human Genetics & Environmental Sciences, Center for Infectious Diseases, School of Public Health, University of Texas Health Sciences Center, Houston, Texas, USA; 3 Division of Infectious Disease, Department of Pediatrics, McGovern Medical School, University of Texas Health Sciences Center, Houston, Texas, USA; 4 Center for Antimicrobial Resistance and Microbial Genomics, McGovern Medical School, University of Texas Health Sciences Center, Houston, Texas, USA; 5 Department of Molecular Microbiology, Washington University School of Medicine, Saint Louis, Missouri, USA; 6 Center for Women’s Infectious Disease Research, Washington University School of Medicine, Saint Louis, Missouri, USA; 7 Division of Plastic and Reconstructive Surgery, Washington University School of Medicine, Saint Louis, Missouri, USA; 8 Division of Surgical Oncology, Washington University School of Medicine, Saint Louis, Missouri, USA; Mayo Foundation for Medical Education and Research, Rochester, Minnesota, USA

**Keywords:** breast microbiome, breast cancer, postantibiotic effect, human microbiome, bioinformatics, clinical trials, clinical methods, microbiome, microbiota

## Abstract

Bacterial infection is the most common complication following staged post-mastectomy breast reconstruction initiated with a tissue expander (TE). To limit bacterial infection, antibiotic irrigation of the surgical site is commonly performed despite little high-quality data to support this practice. We performed a prospective randomized control trial to compare the impact of saline irrigation alone to a triple antibiotic irrigation regimen (1 g cefazolin, 80 mg gentamicin, and 50,000 units of bacitracin in 500 mL of saline) for breast implant surgery. The microbiome in breasts with cancer (*n* = 16) was compared to those without (*n* = 16), as all patients (*n* = 16) had unilateral cancers but bilateral mastectomies (*n* = 32). Biologic and prosthetic specimens procured both at the time of mastectomy and during TE removal months later were analyzed for longitudinal comparison. Outcomes included clinical infection, bacterial abundance, and relative microbiome composition. No patient in either group suffered a reconstructive failure or developed an infection. Triple antibiotic irrigation administered at the time of immediate TE reconstruction did not reduce bacterial abundance or impact microbial diversity relative to saline irrigation at the time of planned exchange. Implanted prosthetic material adopted the microbial composition of the surrounding host tissue. In cancer-naïve breasts, relative to saline, antibiotic irrigation increased bacterial abundance on periprosthetic capsules (*P* = 0.03) and acellular dermal matrices (*P* = 0.04) and altered the microbiota on both. These data show that, relative to saline only, the use of triple antibiotic irrigation in TE breast reconstruction does impact the bacterial abundance and diversity of certain biomaterials from cancer-naïve breasts.

**IMPORTANCE:**

The lifetime risk of breast cancer is ~13% in women and is treated with a mastectomy in ~50% of cases. The majority are reconstructed, usually starting with a tissue expander to help restore the volume for a subsequent permanent breast implant or the women’s own tissues. The biopsychosocial benefits of breast reconstruction, though, can be tempered by a high complication rate of at least 7% but over 30% in some women. Bacterial infection is the most common complication, and can lead to treatment delays, patient physical and emotional distress and escalating health care cost. To limit this risk, plastic surgeons have tried a variety of strategies to limit bacterial infection including irrigating the pocket created after removing the breast implant with antibiotic solutions, but good-quality data are scarce. Herein, we study the value of antibiotics in pocket irrigation using a robust randomized clinical trial design and molecular microbiology approaches.

## INTRODUCTION

The American Society of Plastic Surgeons reports that ~ 193,073 women underwent cosmetic breast augmentation and 83,487 underwent breast reconstruction (BR) with a tissue expander (TE) in 2020 (1). BR is most commonly performed by placing a TE to incrementally stretch soft tissues and restore breast shape. Months later, the TE is exchanged for a more permanent breast implant or an autologous flap (2). TEs are also commonly supported by an acellular dermal matrix (ADM) secured to the chest wall or to the leading edge of the pectoralis major muscle (3). Associated with improved quality of life in appropriately selected patients (4, 5), implant-based BR is also characterized by a significant risk of complications that is largely driven by compromised blood supply or infection (6, 7). Despite efforts to minimize contamination of these devices, such as the use of aseptic environments during implantation, biomaterial that facilitates rapid integration between the prothesis and host cells, as well as perioperative intravenous antibiotics, postoperative oral antibiotics, and suction drains for clearing fluid collections from the surgical site, the risk of infection leading to a failed reconstruction necessitating device explantation ranges from 3.7% to 18.7% of cases (6 – 14).

While most medical devices are implanted in sterile sites, such as hip or knee joints, the breast is a clean-contaminated surgical site populated by bacteria derived from the skin, nipple, and contiguous ductal and glandular tissue, adjacent blood supply, and the translocation of dendritic cells located within the gut (15 – 21). Thus, in addition to acute infection, bacteria also asymptomatically colonize clinically normal breast implants, suggesting that species and/or strain-specific virulence factors, abundance, or interactions with host factors create a homeostatic relationship whose disruption yields pathology (14, 22 – 26). Furthermore, bacterial contamination may also lead to inflammation-related complications like capsular contracture (11, 27 – 29). Notably, additional data also suggest that the diversity of the microbiota within the breast parenchyma may be altered by breast cancer (17 – 19, 30). Thus, limiting bacterial contamination during BR is complex.

One strategy purported to reduce bacterial infection of breast implants or TEs is irrigation of the soft tissue periprosthetic “pocket” with antibiotic solution. A combination of 1 g cefazolin, 80 mg gentamicin, and 50,000 units of bacitracin in 500 cc of normal saline solution is a common breast pocket irrigant (31), although other agents including 10% povidone iodine and stabilized hypochlorous acid are also reported to reduce breast implant infection *in vitro* and clinically (23, 32 – 35). While antibiotics are administered in the perioperative period, breast prostheses indefinitely reside adjacent to bacteria-laden breast tissue. Despite wide clinical adoption of antibiotic pocket irrigation, supportive data are limited. While several reviews have highlighted a knowledge gap and the need for randomized control trials, none exist to assess the clinical impact of antibiotic pocket irrigation on breast implant infection or on the surrounding breast microbiome (36 – 38). Herein, we present a prospective, randomized, controlled trial (PRCT) with a longitudinal study design to investigate the impact of (i) the triple antibiotic pocket irrigant (TAPI): cefazolin, gentamicin, and bacitracin; (ii) breast cancer diagnosis; and (iii) time on the microbiota of the host and implanted tissues in staged TE BR.

## RESULTS

### Patient enrollment and follow-up

**Fig 1:**
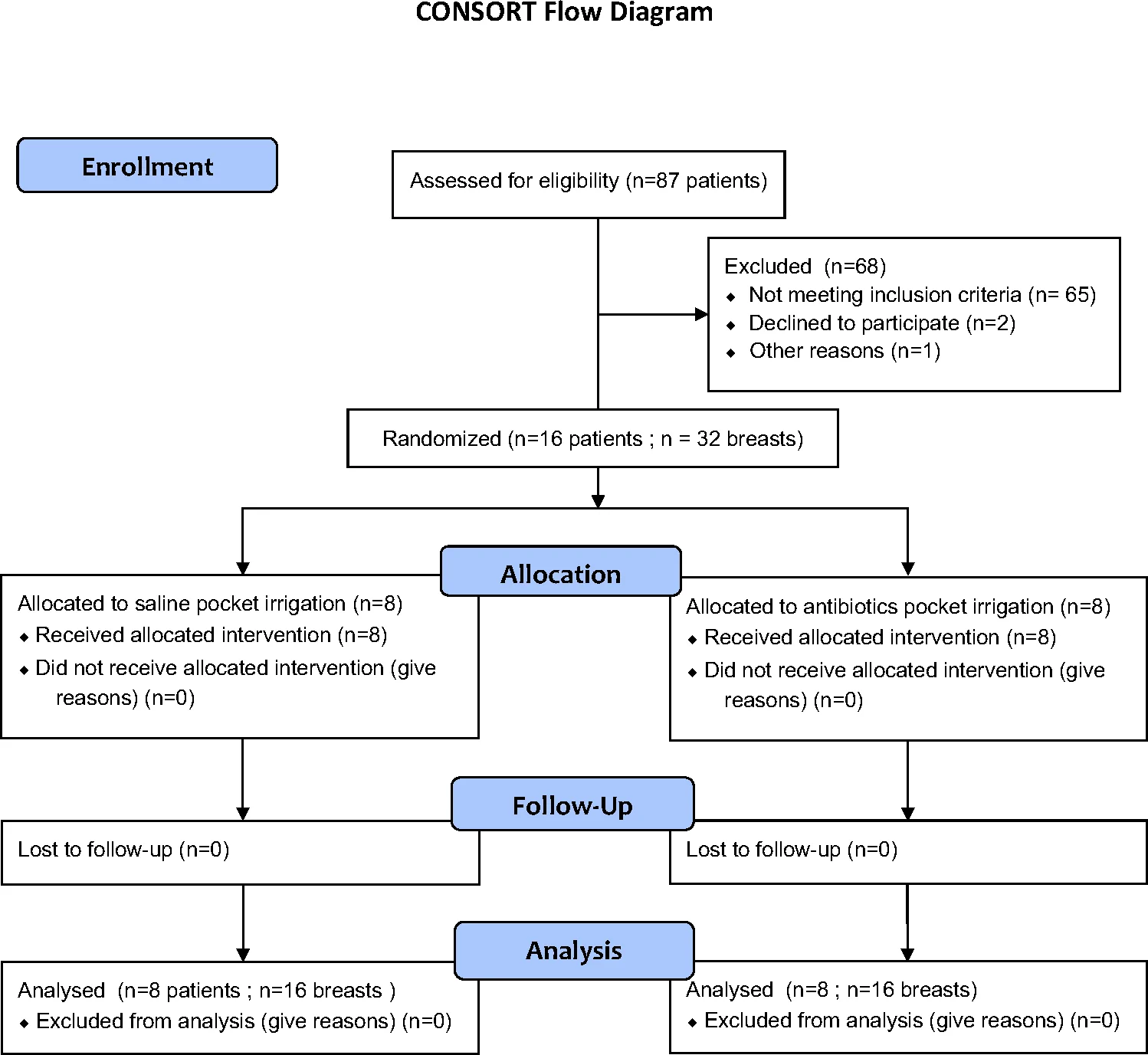
Consort diagram outlining patient enrollment, inclusion and exclusions criteria, randomization to treatment group, and samples analyzed.

**Fig 2:**
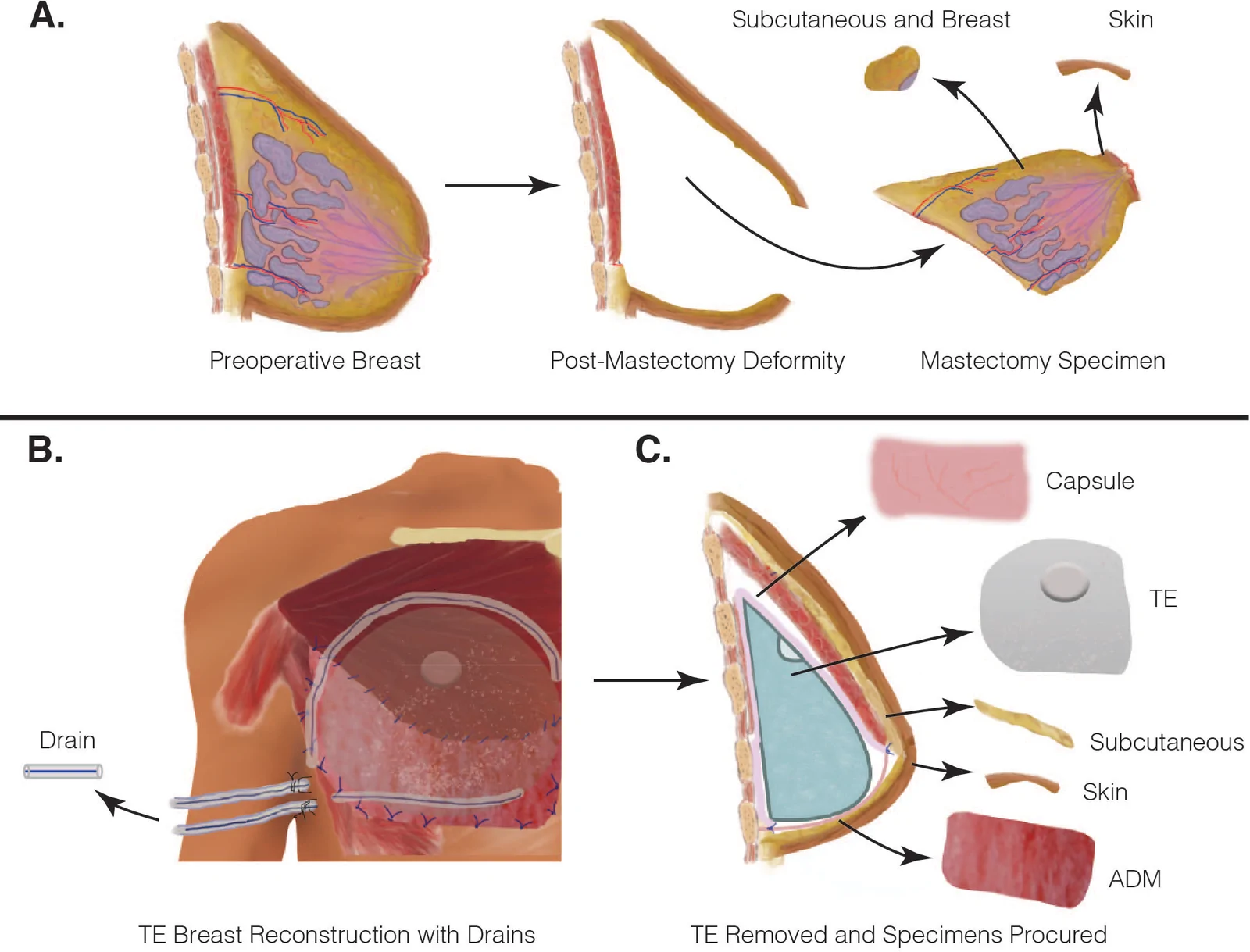
Surgical procedures and specimens analyzed. (**A**) At the time of skin- or nipple-sparing mastectomy, skin and a composite of subcutaneous and breast parenchyma specimens ≥3 sq cm were aseptically collected for analysis. First stage breast reconstruction with a subpectoral textured TE, supplemented with a supportive ADM inferior sling was then undertaken. (**B**) A ≥ 3 cm segment of the indwelling component of a drain from each breast was aseptically collected and stored at the time of removal during an outpatient visit. (**C**) Second stage breast reconstruction, which commenced with procurement of capsule, TE, subcutaneous tissue, skin, and ADM specimens was then performed, and followed by implant or autologous flap reconstruction.

Eighty-seven patients were assessed for study eligibility of which 68 were excluded, with the majority not meeting all inclusion criteria (Fig. 1). Per inclusion criteria, each patient had a unilateral breast cancer, but bilateral mastectomies—therapeutic on the side with breast cancer, prophylactic on the other. This enabled us to study *n* = 16 breasts with breast cancer, and *n* = 16 without (*n* = 32 breasts). Half of the patients (*n* = 8) received TAPI (*n* = 16 breasts). Patient enrollment continued (*n* = 19) until the sixteenth patient underwent Surgery #1. As such, *n* = 16 patients and *n* = 32 breasts comprised the study population (Fig. 1). All patients underwent Surgery #1 between July 2017 and February 2018 and contributed a skin and breast tissue specimen at time of surgery, as well as one drain per breast for analysis in the postoperative period (Fig. 2). Surgery #2 occurred between December 2017 and September 2018 with no loss of follow-up or failures to procure specimens, which included additional skin and breast tissue specimens, as well as TE, ADM, and capsule samples (Fig. 2).

### Baseline characteristics

No statistically significant differences were identified between intervention groups with respect to baseline characteristics including age, smoking, BMI, or relevant medical comorbidities (Table 1). Similarly, cohorts did not differ significantly with respect to oncologic factors like cancer stage or subtype, nor the administration of neoadjuvant or postoperative chemotherapy, or postmastectomy radiation. Two patients, both in the TAPI group, had BRCA2 mutations. One patient, also in the TAPI group, had a PTEN mutation. No other relevant mutations were noted.

**TABLE 1:** Descriptive statistics stratified by pocket irrigation

	Antibiotic pocket irrigation (*n* = 8) *n* (%)	Saline pocket irrigation (*n* = 8) *n* (%)	*P*-value
Age (y)	52.0 ± 10.8	52.6 ± 7.5	0.90
Current Smoking (%)	3 (37.5%)	1 (12.5%)	0.17
BMI (kg/m^2^)	29.3 ± 5.2	27.0 ± 5.3	0.39
Hypertension	3 (37.5%)	2 (25%)	–
Diabetes	0 (0%)	0 (0%)	1.0
Heart disease	0 (0%)	0 (0%)	1.0
COPD	0 (0%)	1 (12.5%)	0.35
No medical comorbidities	5 (62.5%)	8 (100%)	0.08
Cancer type			
Ductal	6 (75%)	7 (87.5%)	–
Lobular	2 (25%)	1 (12.5%)	–
Cancer stage			
0	1 (12.5%)	2 (25%)	–
1	3 (37.5%)	3 (37.5%)	–
2	3 (37.5%)	3 (37.5%)	–
3	1 (12.5%)	0 (0)	–
Adjuvant chemotherapy	1 (12.5%)	3 (37.5%)	0.17
Neoadjuvant chemotherapy	3 (37.5%)	4 (50%)	0.17
Radiation therapy	3 (37.5%)	2 (25%)	0.35

### Clinical outcomes

The primary clinical outcome in our study was reconstructive failure, defined as premature explantation of a tissue expander due to either infection or patient preference. No patient suffered a reconstructive failure. Most patients underwent skin sparing mastectomy in Surgery #1 (Table 2), the remainder nipple-sparing mastectomy, with a mean breast weight of 632.2 ± 296.0 g in the antibiotic versus 690.9 ± 106.3 g in the saline pocket irrigation groups (*P* = 0.46). ADMs consisted of Alloderm RTU (Allergan Aesthetics, Branchburg, NJ, USA) in 62.5% of patients receiving TAPI, and 50% of those receiving the saline control. Cortiva 1 mm (RTI Surgical, Alachua, FL, USA) was otherwise used (39). Time to surgical drain removal, a secondary outcome, was 10.4 ± 7.5 days in the TAPI versus 8.4 ± 1.9 days in the saline pocket irrigation group (*P* = 0.31). While there were no significant differences in the final TE volumes prior to Surgery #2, patients in the saline irrigation group were noted to have significantly less volume placed in their TEs initially (168.8 ± 77.2 vs 236.3 ± 76.2; *P* = 0.02) and subsequently required significantly more office visits (4.8 ± 1.5 vs 3.3 ± 1.2) to fill their TEs and achieve the final volume (*P* = 0.005). No patients developed skin necrosis, and no patients required oral antibiotics beyond our standard treatment protocol. There were no unplanned readmissions.

**TABLE 2:** Surgery #1 details stratified by pocket irrigation

	Antibiotic pocket irrigation (*n* = 16) *n* (%)	Saline pocket irrigation (*n* = 16) *n* (%)	*P*-value
Nipple-sparing	2 (12.5%)	4 (25%)	0.53
Breast weight (g)	632.4 ± 296.0	690.9 ± 106.3	0.46
ADM Alloderm RTU	10 (62.5%)	8 (50%)	0.49
Time to drain removal (days)	10.4 ± 7.5	8.4 ± 1.9	0.31
Initial fill volume (cc)	236.3 ± 76.2	168.8 ± 77.2	0.02
Final expander volume (cc)	476.3 ± 120.2	543.8 ± 161.4	0.19

Patients matriculated to Surgery #2 (Table 3), another secondary outcome, significantly faster in patients receiving TAPI (125.3 ± 46.9 days) versus saline (187.1 ± 50.7 days) pocket irrigation (*P* = 0.001). All non-radiated patients received bilateral silicone breast implants at the time of implant exchange in Surgery #2. No patient developed a pathologic capsular contracture during the study period. For radiated patients, four received bilateral deep inferior epigastric artery perforator (DIEP) microvascular autologous flaps and one received a latissimus flap with silicone implant on the radiated side, and a silicone implant on the non-radiated side (Table 3). No patient suffered a documented infection or reconstructive failure of either their breast implant or autologous flap after at least 1 year of prospective, postoperative follow-up from Surgery #2.

**TABLE 3:** Surgery #2 details stratified by pocket irrigation

	Antibiotic pocket irrigation (*n* = 16)	Saline pocket irrigation (*n* = 16)	*P*-value
Time to OR (days)	125.3 ± 46.9	187.1 ± 50.7	0.001
Tonometer (N/m^2^)	40.9 ± 14.1	33.8 ± 17.8	0.2
Final reconstruction (%)			
Silicone implant	12 (75%)	13 (81.3%)	–
DIEP flap	4 (25%)	2 (12.5%)	–
Latissimus flap with silicone implant	0 (0%)	1 (6.3%)	–
Reconstructive failure	0 (0%)	0 (0%)	–

### Culturable microbes on patient specimens

**Fig 3:**
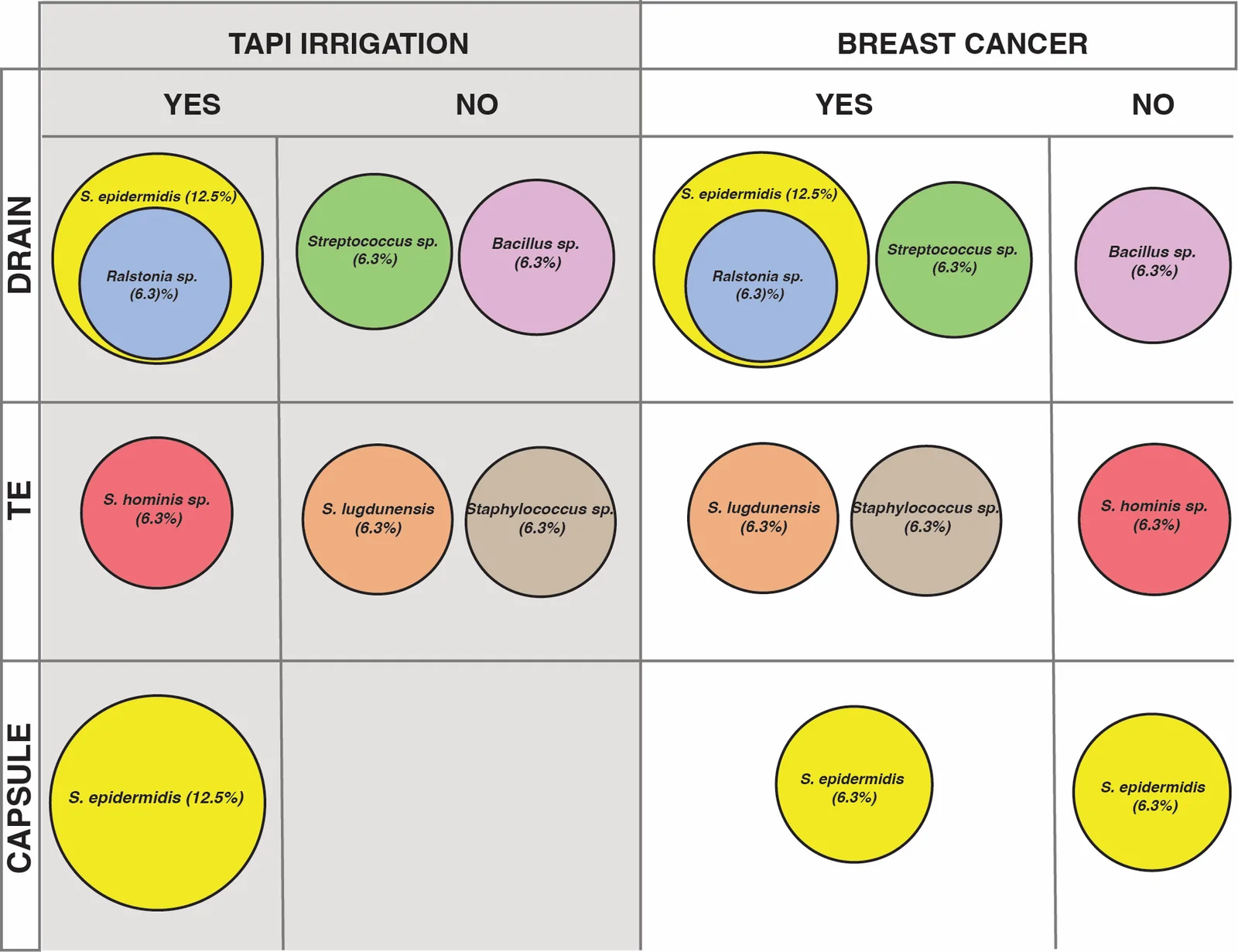
Microbial culture results for each sample type from women with and without the triple antibiotic pocket irrigant (TAPI; gray) and with and without cancer (white). A total of eleven samples from five different patients had culturable bacteria, including three capsules, four tissue expanders (TEs), and four drains. Coagulase-negative staphylococci were the most commonly isolated species among all samples cultured regardless of antibiotic treatment or cancer status. Other Gram-positives, including bacillus and streptococcus spp., were also cultured from a small percentage of drain samples. The Gram-negative *Ralstonia* sp. was cultured from one drain. No Gram-negative bacteria were isolated from TE or capsule samples.

The majority of samples that were cultured did not grow any microbes. However, bacteria could be isolated from ~30% (5/16) of patients’ capsules, TEs, or drains (Fig. 3). Notably, the majority of these microbes (7) were isolated from three women in the TAPI group, while four were isolated from two patients in the saline group (Supplementary File S1). Coagulase negative staphylococci were the most commonly isolated bacterial species, with *S. epidermidis* detected in 2/16 (12%) capsules and 2/16 (12%) drains and other staphylococcal spp., including *S. lugdunensis* and *S. hominis,* each cultured from ~6% of TEs. Additionally, other Gram-positive bacteria, including bacillus and streptococcus spp., were each cultured from ~6% of drain samples. Notably, *Ralstonia* sp. was cultured from one drain. This drain came from a woman in the TAPI group. Overall, the bacteria cultured and isolated from patient samples were similar regardless of cancer status or TAPI.

### Impact of TAPI on bacterial abundance

**Fig 4:**
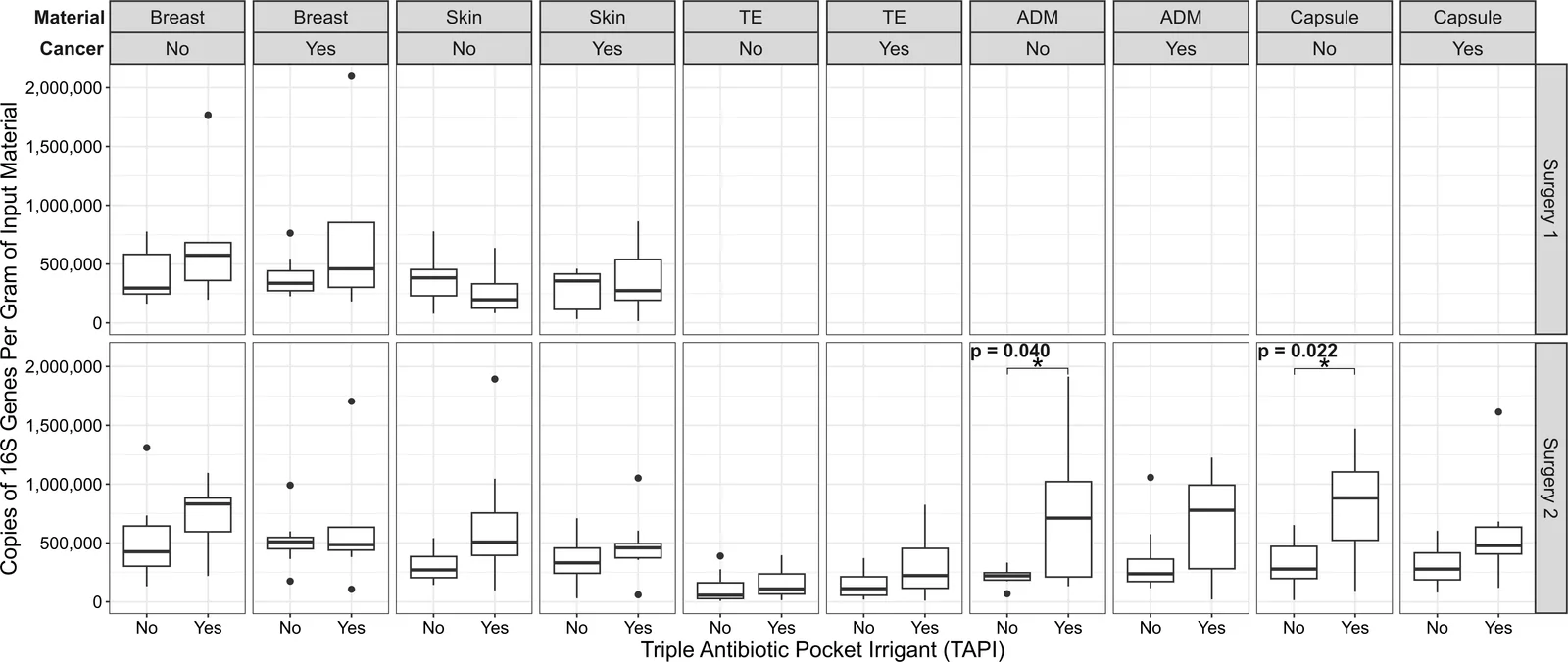
Copies of 16S genes per gram of input material by sample type, antibiotic pocket irrigant exposure, and cancer status at Surgery 1 (top panel) and Surgery 2 (bottom panel). There were no differences observed between breast tissue or skin from antibiotic- and saline-treated women with and without cancer at Surgery #1 or between Surgery #1 and Surgery #2. At Surgery #2, capsules and ADM from women without cancer and with triple antibiotic pocket irrigant (TAPI) treatment displayed significantly more bacteria compared to those in the control group. No differences were observed between any other sample from women with or without cancer or exposed to antibiotic pocket irrigants or not. The Wilcoxon-rank sum test was used to determine statistical significances, with * = *P* < 0.05.

Bacterial abundance among samples collected at Surgery #1 and Surgery #2 was assessed using qPCR and the copy numbers of the 16S rRNA gene present are shown in Fig. 4. There were no statistically significant differences observed in bacterial abundance among breast tissue or skin samples at Surgery #1 from women who received TAPI or saline pocket irrigants or from women with or without cancer. Additionally, there were no significant differences across time (from Surgery #1 to Surgery #2) among these samples. At Surgery #2, capsules and ADMs from women without cancer that received the TAPI displayed significantly higher bacterial abundances compared to capsules from women without cancer that received the saline control (*P* < 0.05). There were no other significant differences detected among any other samples tested.

### Impact of TAPI and breast cancer diagnosis on bacterial ecology

**Fig 5:**
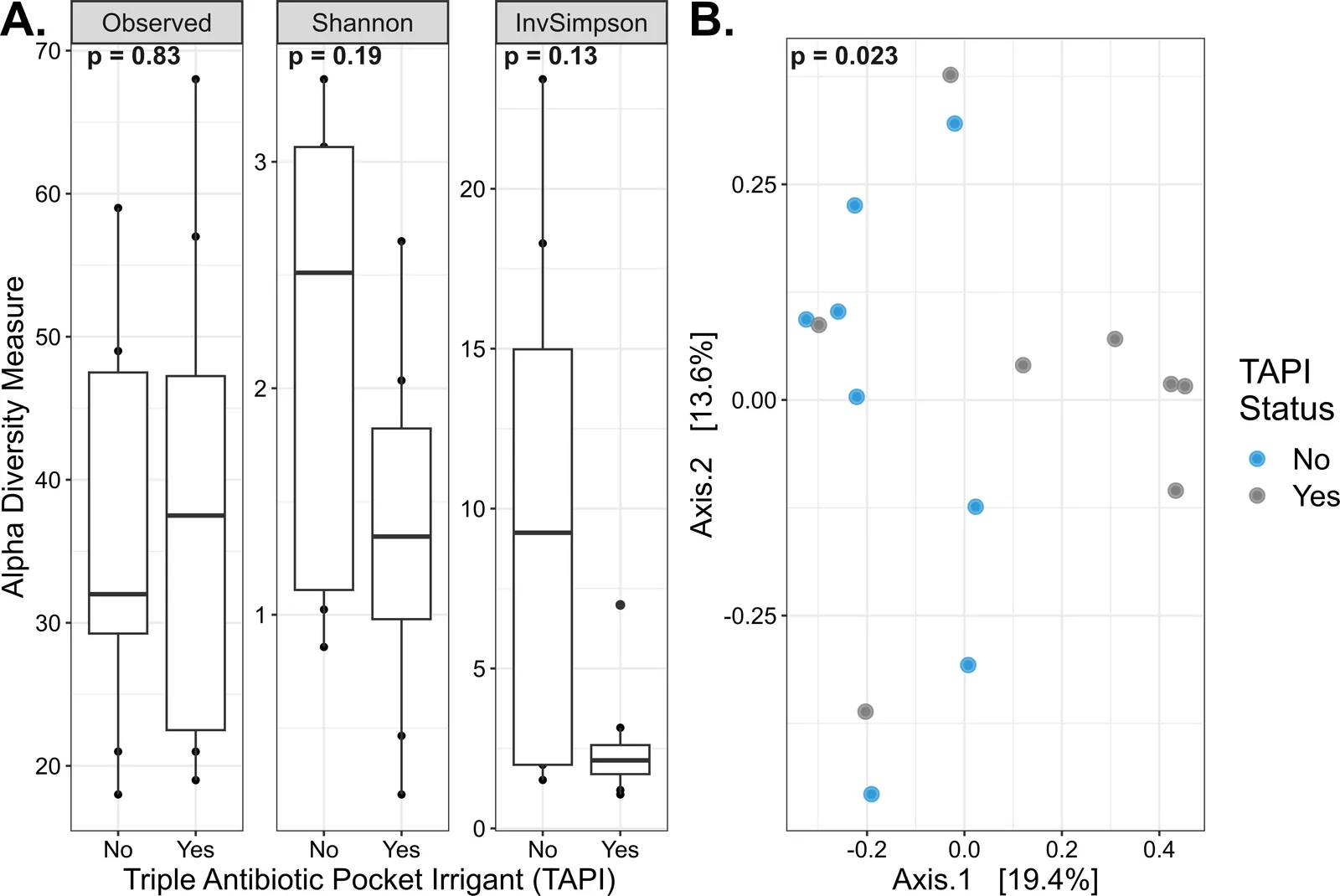
Alpha and beta diversity estimates were assessed for ADM samples from women without cancer, stratified by whether they received or did not receive TAPI. (**A**) Alpha diversity metrics, including Observed, Shannon, and Inverse Simpson (InvSimpson) indexes, indicate there are no statistically significant differences between groups. (**B**) A principal component analysis demonstrates a statistically significant difference in the clustering and centroids when comparing beta diversity of capsule samples by antibiotic status. Additionally, an overall variability of 33.0% was accounted for in axes 1 and 2. The Wilcoxon-rank sum test was used to determine statistical significances, with * = *P* < 0.05.

**Fig 6:**
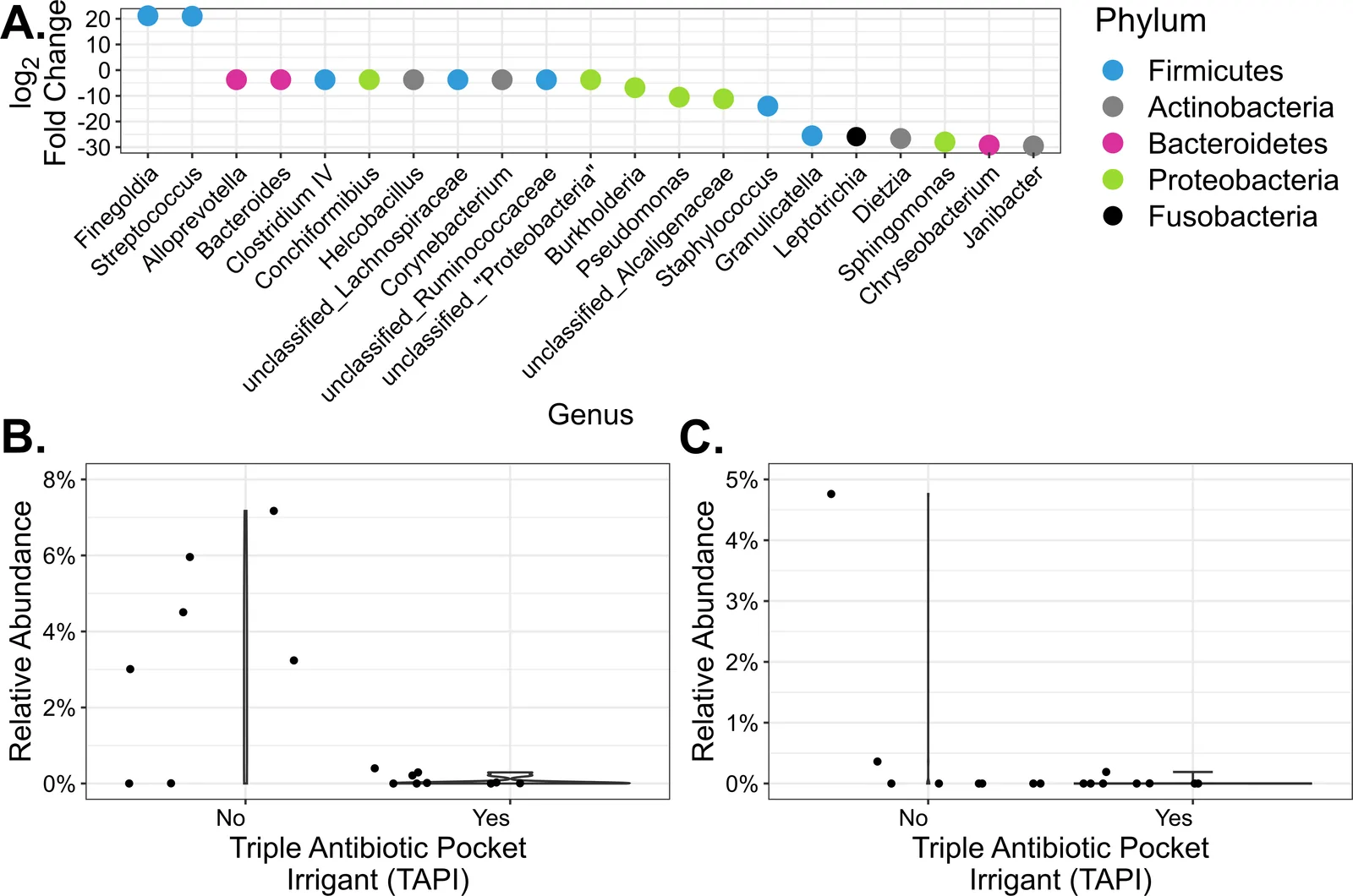
Differentially abundant taxa of ADM samples from women without cancer comparing those with and without TAPI identify taxa whose prevalence (presence/absence) and relative abundance are very low. (**A**) The log2 fold change of taxa identified as differentially abundant. (**B**) Violin plots of the prevalence and the relative abundance of differentially abundant *Burkholderia* OTU demonstrate the presence in all 8 ADMs from women who received the saline control, and in only five ADMs from women who received TAPI. (**C**) Violin plots of the prevalence and the relative abundance of differentially abundant *Staphylococcus* OTU demonstrates the presence in two ADMs from women who received TAPI and two who received the saline control, with only one dominated by these taxa and the rest with relative abundances below 0.5%.

Analysis of 16S rRNA microbiome sequencing indicated that 212 out of 256 patient samples were successfully extracted (*n* = 4 failed extraction), sequenced, and passed quality filtering with at least 5,000 amplicons (*n* = 40 failed filtering requirements) (Supplementary File S1). The majority of samples that failed QC were the low biomass drain samples. A broad range of bacterial species were identified across all samples that passed QC, accounting for 1,957 OTUs. The Observed, Shannon, and Inverse Simpsons’ alpha diversity metrics were assessed. There were no significant differences in alpha diversity metrics among ADM (Fig. 5A), capsule (Fig. S1A), breast skin, tissue, or drain samples (data not shown) from women with and without cancer or with and without TAPI. However, Beta Diversity estimates of ADM (Fig. 5B) and capsules (Fig. S1B) indicated there were differences in the microbial diversity of breasts without cancer who received the TAPI compared to those who did not. While most genus abundances were low, the most abundant genera identified were *Streptococcus*, *Corynebacterium*, *Staphylococcus*, and *Bacillus*, many of which were also cultured from the same samples (Fig. 3 and 6A; Fig. S2A). Differential analyses indicate that the major taxa driving the difference between ADMs (Fig. 6B and C) and capsules (Fig. S2B and S2C) from women without cancer that received TAPI and those that received saline were rare and were driven primarily by signals from individual patients, as each patient looked more like themselves than other patients who received the same treatment or shared cancer status.

### Microbes identified with immunofluorescence

**Fig 7:**
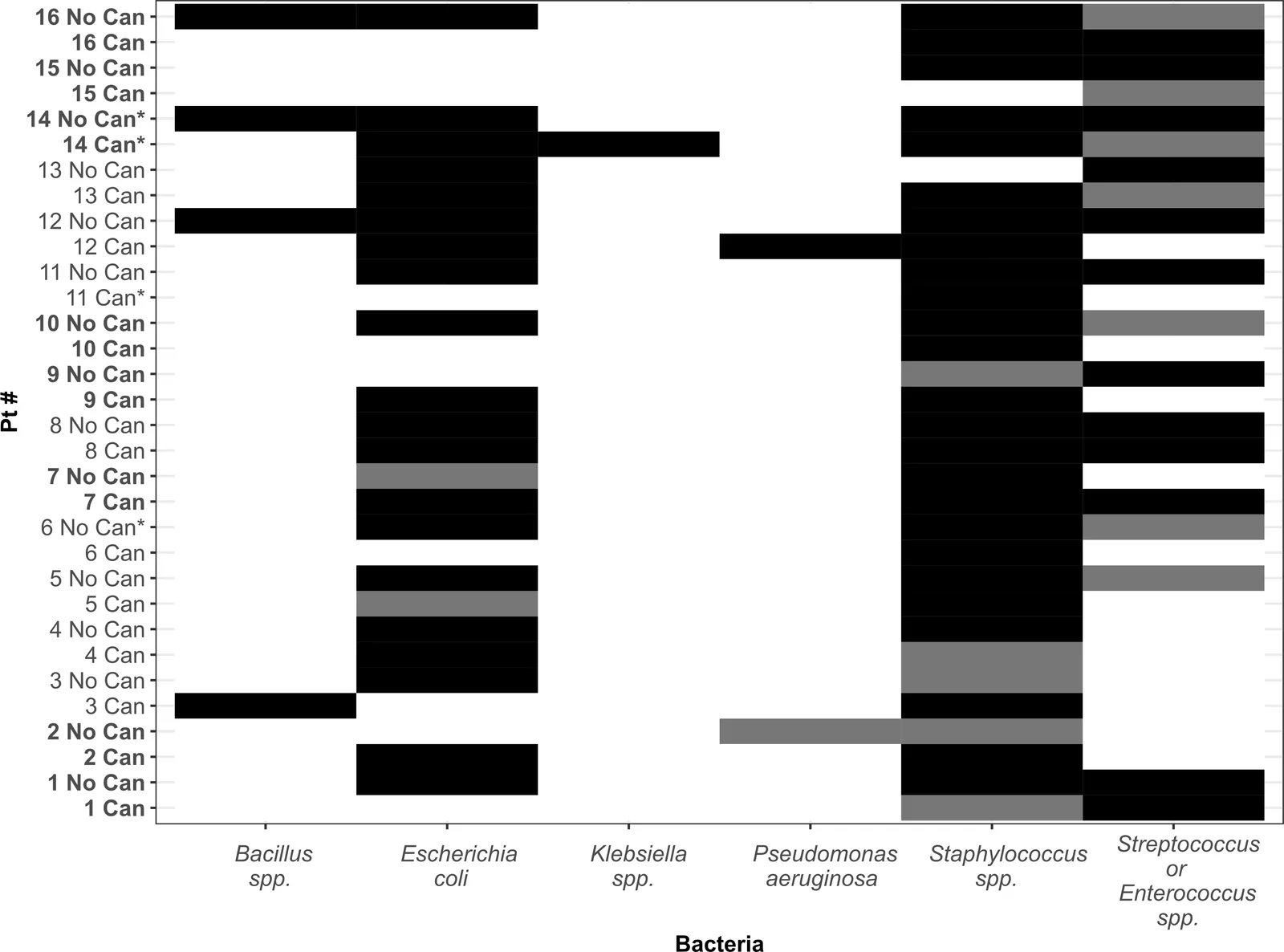
Detection of bacteria via immunofluorescence on tissue expander (TE) samples. TEs were only stained for bacterial species that were detected via culturing or 16S rRNA microbiome sequencing. Staphylococci were the most commonly detected species, followed by other Gram positives, including streptococci, enterococci, and bacilli, and Gram negatives, such as *E. coli*, *P. aeruginosa,* and *Klebsiella* spp. The * indicates samples with culturable microbes, and bolded patient identifiers denote those that received the triple antibiotic irrigant. TEs that were stained for a specific bacterial species and were detected by immunofluorescence staining are denoted with a black bar. TEs that were stained for a specific bacterial species but were not detected via immunofluorescence imaging are indicated with a gray bar. TEs that were not stained for a specific bacterial species are denoted with a white bar.

Immunofluorescence staining was used to visualize bacteria identified via culturing and 16S rRNA microbiome sequencing on the surface of TEs (Fig. 7; Fig. S3). Commercially available antibodies were used to detect bacteria including staphylococcal, streptococcal, enterococcal, *Bacillus*, *Klebsiella*, *Escherichia*, and *Pseudomonas* spp. Bacteria could be detected via immunofluorescence on all but one (15/16) of the TEs stained, with the majority containing staphylococcal species. This corresponds with the culture and 16S rRNA microbiome sequencing data.

## DISCUSSION

With a lifetime risk of 1 in 8, breast cancer is the second most common cancer among women (40). Mastectomy rates, whether for therapeutic or prophylactic purposes, have increased (41 – 44), as have the rates of immediate BR with breast implants or TEs (45). When contemplating BR, women must weigh the real risk of complications (46), especially infection and implant explantation (47). Acute implant infection is an ongoing challenge for plastic surgeons as they seek strategies to mitigate the impact of the breast and skin microbiota during the placement of a permanent breast implant into the clean-contaminated surgical site immediately after a mastectomy (17, 21). The vast majority of plastic surgeons use antibiotic pocket irrigation with either reconstructive or aesthetic breast implants (48, 49). This strategy is largely based on guidance that recommends the use of antibiotic pocket irrigation to reduce bacterial load, thereby minimizing not only acute implant infection, but also the development of the latent complication, capsular contracture. Capsular contracture can form around breast implants even a decade or more after placement, and has been linked to increased bacterial abundance on breast implants (27, 28, 50 – 55). However, the nature of this association—whether commensal, opportunistic, or causative—remains under investigation. Notably, many studies assessing the efficacy of antibiotic pocket irrigants rely on *in vitro* analyses or clinical studies that are not well controlled nor employ a randomized prospective study design (31, 34, 56). Yet, bacterial mitigation strategies using antibiotic pocket irrigation are now recommended to prevent these long-term sequelae, including capsular contracture (57 – 60). Herein, our randomized and longitudinal study design enabled assessment of the microbiome over time among skin and subcutaneous breast tissue samples. Notably, no significant differences were detected in bacterial abundance or alpha diversity among any of the skin and tissue samples at the time of mastectomy compared to ~5 mo later when devices were exchanged (Fig. 4). Furthermore, additional analyses show that early intervention with TAPI does not significantly reduce bacterial abundance or impact alpha diversity metrics for the majority of samples (skin, tissue, and TEs) months later compared to a saline irrigation control, thus refuting this broadly implemented (48, 49, 61), but minimally investigated, heuristic.

Additionally, the results from this study indicate that when subdivided by the presence of a cancer diagnosis or use of TAPI, the bacterial load in capsules and ADMs in cancer naïve breasts was significantly increased in those that received TAPI relative to saline (Fig. 4). Notably, subinhibitory concentrations of antibiotics can induce the formation of communities, such as aggregates, agglutinates, or biofilms, by resident planktonic bacteria potentially derived from the breast. Furthermore, host proteins like fibrinogen and collagens, readily available from serum, capsule, and ADM, may potentiate bacterial recalcitrance (23, 24, 26, 62 – 64). Notably, our recent report indicates that TAPI, in addition to enhancing biofilm formation of *S. aureus* breast implant infection isolates *in vitro,* also had no effect on *S. aureus* bacterial load in a mouse model of breast implant infection compared to a saline irrigant control (65). Finally, bacteria exposed to antibiotics may also rapidly evolve or acquire genetic mutations that confer antibiotic resistance to promote survival (66). Together, these mechanisms may facilitate bacterial contamination of breast implants despite initial management with TAPI. Additionally, these conditions may be more permissive in cancer naïve than cancer positive capsules leading to a statistically significant increase in bacterial load.

The results from this study also indicate that capsules and ADMs from women without cancer that received TAPI have different centroids of the clusters (Fig. S1; Fig. 5B) and differentially abundant taxa when comparing the clusters to those that received the saline irrigant control. These differences were mostly driven by rare and/or very low abundance taxa (Fig. 6; Fig. S2). This difference may be related to the significantly lower bacterial biomass observed compared to all other samples (Fig. 3). Lower abundance microbial communities on capsules and ADMs may lack the latitude to recover after exposure to antibiotics, relative to all other tissue samples, which demonstrated greater abundance and potentially less vulnerability (67, 68). Notably, commensal dysbiosis in the breast and gut may shift the elaboration of bacterial metabolites and affect the regulation of inflammatory processes leading to breast and colorectal cancer, type I diabetes, and many other diseases (68 – 71). While the clinical impact of reducing richness of microbial communities on capsules and ADMs is unknown, the microbiota identified on samples from saline control pockets in this study could be considered commensal, and the use of TAPI may lead to dysbiosis.

While little is known about the time course of breast implant bacterial contamination in the clinical setting, as devices or capsules are only accessible during a planned exchange of a TE or when there is a complication necessitating surgical intervention, we highlight the limitations of relying on standard culture techniques, which are broadly employed to confirm breast implant infection or colonization (25, 26, 28, 55, 72, 73). Specifically, when a multimodal approach with progressively more sensitive techniques, such as 16S sequencing or microscopy, are employed, evidence of bacteria can be identified on virtually all samples (capsules, TEs, ADMs, skin, and tissue). By contrast, culture techniques, even when accompanied by sonication, only identified bacteria from 30% of patients, and most commonly from the drain, capsule, or TE. Despite evidence for bacterial contamination of TEs, ADMs, and capsules, no patient in our series developed a clinically relevant infection. These data support reports indicating that only a minority of women develop clinically relevant pathology. In particular, an 8.9% surgical site infection rate in the first 90 days following post-mastectomy BR is reported in a cohort of 7,655 women (47), but infection rates of up to 34% are reported in some series (74). The mean time from TE placement (Surgery #1) to exchange (Surgery #2) was ≥125 days in our study (Table 3), varying based on requirements for intervening adjuvant chemotherapy or radiation in many cases. This also provided ample time for most clinical infections to manifest (11, 12). These data suggest that while clinical infections are relatively common, TEs, ADMs, and capsules can harbor commensal bacteria, likely existing as biofilms. Thus, the mere presence of bacteria does not portend clinical sequela.

The widespread identification of bacteria on TEs, ADMs, and capsules in this study merits consideration, especially considering that most implanted devices, such as knee and hip joints, are sterile; while others, such as suprapubic catheters, become chronically, asymptomatically colonized with multiple pathogens (75 – 77). Furthermore, *in vitro* studies assessing specific antibiotics, doses, and durations of exposure to various bacteria that commonly infect breast implants do so under the assumption that breast implants are more similar to knee joints as opposed to suprapubic catheters (23, 58, 59, 78). However, this study suggests breasts and the devices within them may in fact maintain stable microbial communities. While it is possible that the administration of TAPI in the short term affects the composition of these communities, the latitude and subsequent recovery time between Surgery #1 and #2 afforded the microbial communities sufficient time to overcome a pulse perturbation (67, 68, 79). Furthermore, during surgery antibiotics have an impact for only a finite period of time. Thereafter, if bacteria translocate from the skin, remaining subcutaneous tissues, drain site, or the gut through hematogenous spread, microbial colonization can be reestablished, particularly over the course of months where a TE, or years where a permanent breast implant, remains *in situ* (18, 68, 69, 80).

Potentially complicating the effective management of breast implant infection further, several studies report the breast microbiome is altered with breast cancer (17, 19, 68, 69, 81). While, in this trial, we did not detect a significant difference within the composition of the breast microbiota between the side with cancer and contralateral controls, these findings are consistent with previous work showing similar levels of richness and diversity between specimens associated with breast cancer and normal controls within individuals (19, 82). Discrepancies between studies identifying a distinct breast microbial signature in breast cancer and those that do not (17, 30) may be related to several factors. One factor may be the proximity of the assessed tissues in relation to the malignancy itself. Studies assessing the microbiome of breast tumors or nearby tissue (within ~5 cm) indicate that breast tissue near tumors (within individuals) as well as benign breast tissue from healthy patients (between individuals), respectively, display similar microbial profiles (82, 83). In contrast, a separate study showed the tissue adjacent to post-resection cavities of malignancies in patients with breast cancer displayed enrichment of taxa with lower abundance compared to breast tissue from patients undergoing surgery for benign breast disease (17). In our study, mastectomies were exclusively performed and breast tissues from the side with cancer were typically sampled ≥3 cm from the known gross tumor margin to avoid disrupting the assessment of margins. Following mastectomy, at the time of Surgery #2, breast specimens were taken from lateral parenchymal tissue. Patients served as their own controls, comparing the side with cancer to the contralateral breast. Furthermore, breast cancer stage or histologic subtype may also contribute to the discrepancy. Although results have been inconsistent, some studies suggest bacterial DNA load in tumor tissue and advanced-stage breast cancers is reduced compared with early staged cancers or normal tissues (19). In our study, the majority of breast cancers were early stage invasive ductal cancers, consistent with the most common cohort of women undergoing post-mastectomy BR (84). Lastly, in the gut, radiation is known to limit bacterial diversity leading to dysbiosis (85, 86). In breasts, radiation causes substantial soft tissue compromise and has a profound impact on BR failure rates (12, 46). Importantly, 37.5% of breasts treated with TAPI, and 25% with saline were subject to post-mastectomy radiotherapy prior to Surgery #2. Thus, it is feasible that radiation-induced dysbiosis may have confounded any impact of the TAPI. However, detailed characterization of the impact of radiation on the breast microbiome remains unknown.

Despite the inherent strengths of a prospective, randomized, controlled trial, our study has several notable limitations. The trial was underpowered to either detect more subtle differences in microbial diversity or to perform subset analyses on a complex population of women treated for breast cancer. In the face of modest power and numerous comparisons, we may have observed trends rather than significance where differences between groups were sufficiently subtle, or our statistically significant findings may have occurred by chance. Additionally, while clinically favorable, a lack of infections prevented us from studying potential differences in the microbiota of these patients. We also used patients as their own controls when comparing breasts with and without cancer. However, it is feasible that dysbiosis of the parenchyma occurs at a patient- rather than breast-level in women with cancer. If this is the case, variation in microbial diversity between individual patients would make characterizing a particular microbial signature common to all breast cancer patients extremely challenging. Further, while we characterized microbial communities over time, several sample types, and conditions of malignancy and antibiotic exposure employing metataxonomic, imaging, and culture techniques with clinical translation, future directions should employ functional analyses with metagenomics and metabolomics. In addition, it is feasible that the evolution of reconstructive techniques and devices to include prepectoral reconstruction (87), a shift away from macrotextured implants (88, 89), and alternative antiseptics and antibiotics used in pocket irrigation may also impact the interaction between bacteria, host, and device (33, 56, 90). Lastly, while all of our study specimens were procured in a sterile operating setting using chlorhexidine skin preparation and perioperative intravenous antibiotics followed by immediate transfer from the patient to 200 proof ethanol in the operating room, it is feasible that some became contaminated during device retrieval (91). However, at least one TE had no detectable microbes via culturing or immunofluorescence staining. Additionally, a recent report found that external contamination of surgically excised breast tissue was not significantly different from samples retrieved through core needle biopsies (82). In addition, TE, ADMs, and capsules are characterized by a lower overall bacterial biomass and can be sporadically contaminated, thus appropriate negative controls, including negative extraction controls for 16S rRNA sequencing, are essential and were used in the study.

### Conclusions

TAPI did not reduce bacterial abundance or impact microbial diversity relative to saline irrigation in breasts with cancer undergoing BR with TEs after mastectomy. In breasts without cancer, TAPI significantly increased bacterial abundance in capsules and ADMs, while also resulting in changes in the microbial composition. We did not identify a lasting perturbation of the diversity and composition of bacteria following cancer treatment or TAPI in breast skin or tissue. Additionally, the implanted TEs, capsules, and ADMs adopted the microbial composition of the surrounding host tissues whether or not cancer or TAPI were present. These data question the value of antibiotic pocket irrigation in the face of a resilient microbial ecosystem. Recognizing an unacceptably high rate of infection around breast prostheses, a more granular and precise knowledge of the functional microbiome is required to personalize treatment for the prevention of infection in higher-risk women.

## MATERIALS AND METHODS

### Study design

We performed a double-blind longitudinal, PRCT to investigate the impact of equal volumes of standard antibiotic pocket irrigation versus normal saline irrigation on bacterial contamination and clinical outcomes in staged TE BR. Both patients and researchers were blinded to the administration of saline versus antibiotic pocket irrigation. Every woman underwent two surgical procedures during the study period. For Surgery #1, every woman underwent a unilateral therapeutic mastectomy for breast cancer and contralateral prophylactic mastectomy and was reconstructed with bilateral subpectoralis major muscle TEs with ADMs for inferior pole soft tissue support (92).

### Enrollment and randomization

Women with a unilateral breast cancer who opted to have bilateral mastectomy with one surgical oncologist (JM) with immediate implant-based BR (TM/MT) for Surgery #1 were screened for enrollment. Women with a known allergy to any of the study antibiotics, body mass index ≥35 kg/m^2^, refused study participation, or favored a pre-pectoral, direct-to-implant, or autologous flap reconstruction were excluded. Enrolled women were randomized to receive saline versus antibiotic pocket irrigation as determined by the randomizer.org web-based application (Fig. 1).

### Study intervention

Patients received either 500 cc of normal saline, or antibiotic pocket irrigation consisting of 1 g of cefazolin, 80 mg of gentamicin, and 50,000 units of bacitracin in 500 cc of normal saline. Pocket irrigation was administered for 2 minutes following mastectomy with TE and ADM reconstruction during Surgery #1, after which the surgical incision was closed without aspirating the irrigant.

### Surgery #1 surgical technique, perioperative management and follow-up

Surgical approach was fully standardized including the preoperative decontamination protocol (chlorhexidine wipes), criteria for nipple-preservation, and facility. For Surgery #1, the skin was prepared with 10% povidone iodine solution in the operating room. All patients underwent bilateral skin-sparing or nipple-sparing mastectomies (Fig. 2A) with immediate placement of a macrotextured TE (Allergan, Irvine, CA, USA) (Fig. 2B). TEs were placed deep to the caudally disinserted pectoralis major muscle, and supported with a 128 sq cm sheet of ADM along the lower pole of the reconstruction randomized between Alloderm RTU (Allergan Inc, Irvine, CA, USA) or Cortiva thin (RTI Surgical, Alachua, FL, USA) for both plastic surgeons. Suction drains, including a 10 Fr deep, and a 15 Fr superficial to the pectoralis major muscle were placed in each breast and stabilized at the skin with a chlorhexidine-impregnated patch (BioPatch, Ethicon Inc, Somerville, NJ, USA).

All patients received 2 g of cefazolin intravenously immediately prior to Surgery #1 and then 1 g of cefazolin postoperatively every 8 hours for three doses. Patients were discharged on the first postoperative day and were maintained on oral antibiotics (250 mg of cephalexin every 6 hours) while surgical drains were *in situ*. Each surgical drain was removed when output dropped to <30 cc per day for two consecutive days. Routine follow-up occurred 7 and 21 days postoperatively in addition to visits for drain removal per the above criteria.

### Surgery #2 procedures and decision-making

Clinically, Surgery #2 was a planned procedure to exchange the TEs for either a breast implant or autologous flap reconstruction. Experimentally, Surgery #2 enabled the study team to perform excisional biopsies of implanted TEs and ADMs as well as autologous skin, subcutaneous tissue, and periprosthetic capsules without clinical consequence. Reconstruction occurred after study biopsies were performed and were guided by a risk-sensitive and patient preference concordant shared-decision making process (5).

### Clinical outcomes

Time to drain removal, skin necrosis, infection, unplanned readmission, unplanned antibiotic use beyond postoperative drain removal, reconstructive failure defined as unplanned TE explantation or exploration, administration of neoadjuvant or adjuvant chemotherapy or radiation and the type of reconstruction in Surgery #2 were all prospectively recorded.

### Tissue and prosthetic material procurement

Biopsies were consistently obtained from each breast for every study patient. During Surgery #1, and prior to the conclusion of mastectomy, ≥3 sq cm biopsies of the skin and underlying breast parenchyma were obtained (Fig. 2A) at least 3 cm away from a known tumor margin to avoid compromising pathologic assessment. After Surgery #1, one suction drain was aseptically collected in the plastic surgery outpatient clinic at the time of removal and only the portion within the breast tissue was retained for future analyses (Fig. 2B). All patients matriculated, uneventfully, to Surgery #2 where biopsies from the skin (≥3 sq cm), ADM (≥3 sq cm), TE (≥30 sq cm), capsule (≥3 sq cm), and subcutaneous tissues (≥ 3 sq cm) were obtained (Fig. 2C). Immediately upon removal, specimens intended for DNA extraction and 16S rRNA sequencing were stored in 200 proof ethanol (Fisher Scientific; BP2818500), while remaining samples were immediately placed in 4% phosphate buffered solution (PBS) for bacterial culturing and immunofluorescence staining as previously described (25, 93) (also see Bacterial Culture, Bacterial Identification, Immunofluorescence Sample Processing, and Microbiome Sample Processing sections below).

### Bacterial culture

Each specimen (skin, breast tissue, TE, ADM, and capsule) in PBS was cut into three pieces, and the largest piece (1–25 sq cm depending on the specimen) was processed for immunofluorescence (see Immunofluorescence Sample Processing section below). The two smaller pieces (~5 sq mm) were weighed and assessed for bacterial growth in two different ways. Briefly, the first piece was placed in 1× PBS, sonicated for 10 minutes, vortexed briefly, and serial dilutions were plated on brain heart infusion (BHI) agar (VWR; 90003-040), as previously described (25, 26). Plates were incubated at 37°C for 24–48 h under both aerobic and anaerobic conditions. Colony forming units (CFUs) for each morphologically different colony were assessed and one colony of each morphology was stored at −80°C until identification by 16S sequencing (see Bacterial Identification section below). The second piece was placed in BHI broth and grown for 24–48 hours at 37°C under shaking conditions. Cultures with visible bacterial growth were re-struck on BHI agar plates for single colonies. Each morphologically different colony was stored until identified by 16S sequencing (see Bacterial Identification section below). The remaining 1 mL of the initial heterogeneous culture was stored at −80°C.

### Bacterial identification

To identify the bacteria cultured from each sample, 16S sequencing was utilized as previously described (25, 93). Briefly, each morphologically different bacterial isolate was struck onto a BHI agar plate from the frozen stock. DNA was extracted from a single colony using the DNeasy PowerBioflm kit (Qiagen; Cat # 24000-50), according to the manufacturers’ protocol. For the bead beating step, the MP Biomedicals FastPrep-24 Classic Instrument (MP Biomedicals; Cat # MP116004500) was used. Bacterial identification was performed with 16S Sanger Sequencing using the V1-V3 primers shown in Table S1. The National Center for Biotechnology Information BLAST database was used to align sequences with known bacteria (94).

### Immunofluorescence sample processing

The largest section of patient TEs (5–25 sq cm) was fixed in 10% neutral buffered formalin (Sigma Aldrich; HT501128-4L), shaken overnight at 4°C, washed with 1× PBS, and stored in 1× PBS at 4°C, as previously described (25, 93). Fixed samples from each patient were divided into smaller sections for immunofluorescence staining of bacteria identified via culture-based and microbiome results. Briefly, TE sections were incubated in blocking buffer (1.5% bovine serum albumin and 0.1% sodium azide in 1× PBS) overnight at 4°C and then washed with PBS-T (0.05% Tween-20 in 1× PBS). Primary antibodies (Table S2) were diluted ~1:500 in dilution buffer (0.05% Tween, 0.1% bovine serum albumin, and 0.5% methyl alpha-D-mannopyranoside in 1× PBS) and incubated with the TEs for 2 hours. Following incubation, TEs were washed with PBS-T and incubated with secondary antibodies (Table 2) diluted 1:10,000 in dilution buffer for 1 hour. TEs were washed again with PBS-T (three times for 5 minutes) and air dried for >48 hours. The Odyssey Imaging System (LI-COR Biosciences; Cat# B446) was used to detect infrared signal. Negative controls included a small piece of each TE that was treated identically as described above; however, the primary antibodies were excluded.

### Microbiome sample processing

Each sample (skin, breast tissue, TE, ADM, and capsule) was stored in ethanol at −80°C until sample processing (25, 93). Briefly, DNA was extracted from a piece of each specimen (~200 mg) using the DNeasy PowerBioflm kit (Qiagen; Cat # 24000-50); according to the manufacturers’ protocol. The MP Biomedicals FastPrep-24 Classic Instrument (MP Biomedicals; Cat # MP116004500) was used for the bead beating step. A negative extraction control was included with each sample extraction batch to account for any possible contaminants, including those in the kit. Extracted DNA was stored at −20°C until Illumina sequencing was performed.

### Microbiome sequencing and data processing

16S rRNA microbiome sequencing was performed as described previously (25). Briefly, the V1-V3 region of the 16S rRNA gene was amplified for each sample utilizing unique dual barcodes and sequenced using an Illumina MiSeq with a 2 × 300 v3 sequencing kit (Illumina; Cat # MS-102-3003). Sequencing data were processed using the following methods: trimmomatic to remove the 16S primer sequences (95); PEAR to assemble amplicons from paired sequences (96); BMTagger to remove human DNA sequencing data (97); USEARCH v11.0.667 to generate 97% operational taxonomic units (OTUs) (98); and the RDP Classifier to identify taxonomy of each OTU (99). Successful samples had more than 5,000 amplicons following processing (Supplementary File S1).

### 16S rRNA qPCR quantification

To quantify the number of bacteria within each sample, quantitative PCR (qPCR) was performed as previously described (25). Briefly, qPCR was performed through an adapted version of the BactQuant protocol, which quantified the relative number of bacteria within each tissue type (100). The same 16S primers, amplification conditions, and analysis methods were employed, with the exception that SybrGreen was used in place of the Taqman probe. All samples were run in a triplicate, with positive and negative controls, and the *Escherichia coli* known standard curve, with gene copies ranging from 10^2^ to 10^7^. The samples were standardized by calculating the copies of 16S gene per gram of input material.

### Statistics

A sample size calculation was not performed since data to inform the effect size of antibiotic pocket irrigation or breast cancer diagnosis on clinical infection rates, or microbial composition and diversity, in TE BR do not exist. Statistical assessments of diversity by antibiotic usage and cancer included richness estimates, Shannon diversity, and the Inverse Simpson tests. We also performed beta diversity assessments, including PERMANOVA and Adonis tests. The Phyloseq package was used to assess alpha diversity for each sample using the observed diversity. All analyses were completed with R version 4.1.1.

## Data Availability

Illumina sequencing data with potential host contamination, as well as assembled amplicons used in the analyses of this manuscript, can be found under NCBI BioProject PRJNA893828.

## SUPPLEMENTAL MATERIAL

File S1 spectrum.01430-23-s0001.xlsx Patient sample information including sample type, collection time point, antibiotic exposure, cancer status, microbial culture results, and sequencing.

Supplemental material spectrum.01430-23-s0002.docx Tables S1 and S2 and Fig. S1 to S3.

ASM does not own the copyrights to Supplemental Material that may be linked to, or accessed through, an article. The authors have granted ASM a non-exclusive, world-wide license to publish the Supplemental Material files. Please contact the corresponding author directly for reuse.
