# Supplementary material for: A prospective randomized clinical trial to assess antibiotic pocket irrigation on tissue expander breast reconstruction
Source: Microbiol Spectr. 2023 Sep 27;11(5):e01430-23. doi: 10.1128/spectrum.01430-23 (PMC10581127; doi:10.1128/spectrum.01430-23)
Supplement: Supplemental material — Tables S1 and S2 and Fig. S1 to S3. [file spectrum.01430-23-s0002.docx]

**SUPPLEMENTAL MATERIALS**

**SUPPLEMENTAL TABLES**

**Table S1**. 16S sequencing primers used for bacterial identification.

Primer name Primer sequence (5’ to 3’) size

16S V3_334F CCAGACTCCTACGGGAGGCAG

500bp

16S (806R) Universal GGACTACHVGGGTWTCTAAT

**Table S2**. Antibodies used for Immunofluorescence Staining

| **Primary antibody** | **Genus detected** | **Supplier** | **cat #** | **reference** |
| --- | --- | --- | --- | --- |
| rabbit | *Staphylococcus aureus* | Sigma-Alderich | P3775 | Walker et al.(29) |
| mouse | *Staphylococcus* spp. | ThermoFisher Scientific | MA1-35788 | Walker et al.(29) |
| rabbit | *Pseudomonas* spp. | Abcam | ab68538 | Walker et al.(29) |
| rabbit | *E. coli* | US biologics | E3500-04K | Walker et al.(28) |
| rabbit | *Enterococcus* spp. | ThermoFisher Scientific | PA1-73120 | Walker et al.(29) |
| rabbit | *Bacillus* spp. | Abcam | Ab20556 | N/A |
| rabbit | *Klebsiella* spp. | Virostat | N/A | Walker et al.(29) and (101) |
| rabbit | *Streptococcus* spp. | Lee Laboratories | N/A | Walker et al.(29) and  Flores-Mireles et al.(89) |
|  |  |  |  |  |
| **Secondary antibody** | **Genus detected** | **Supplier** | **cat #** | **reference** |
| IRDye 680LT Donkey | rabbit | LI-COR biosciences | 926-68023 | Walker et al.(28) |
| IRDye 800CW Donkey | mouse | LI-COR biosciences | 926-32212 | Walker et al.(28) |

**SUPPLEMENTAL FIGURES: Capsule**

**
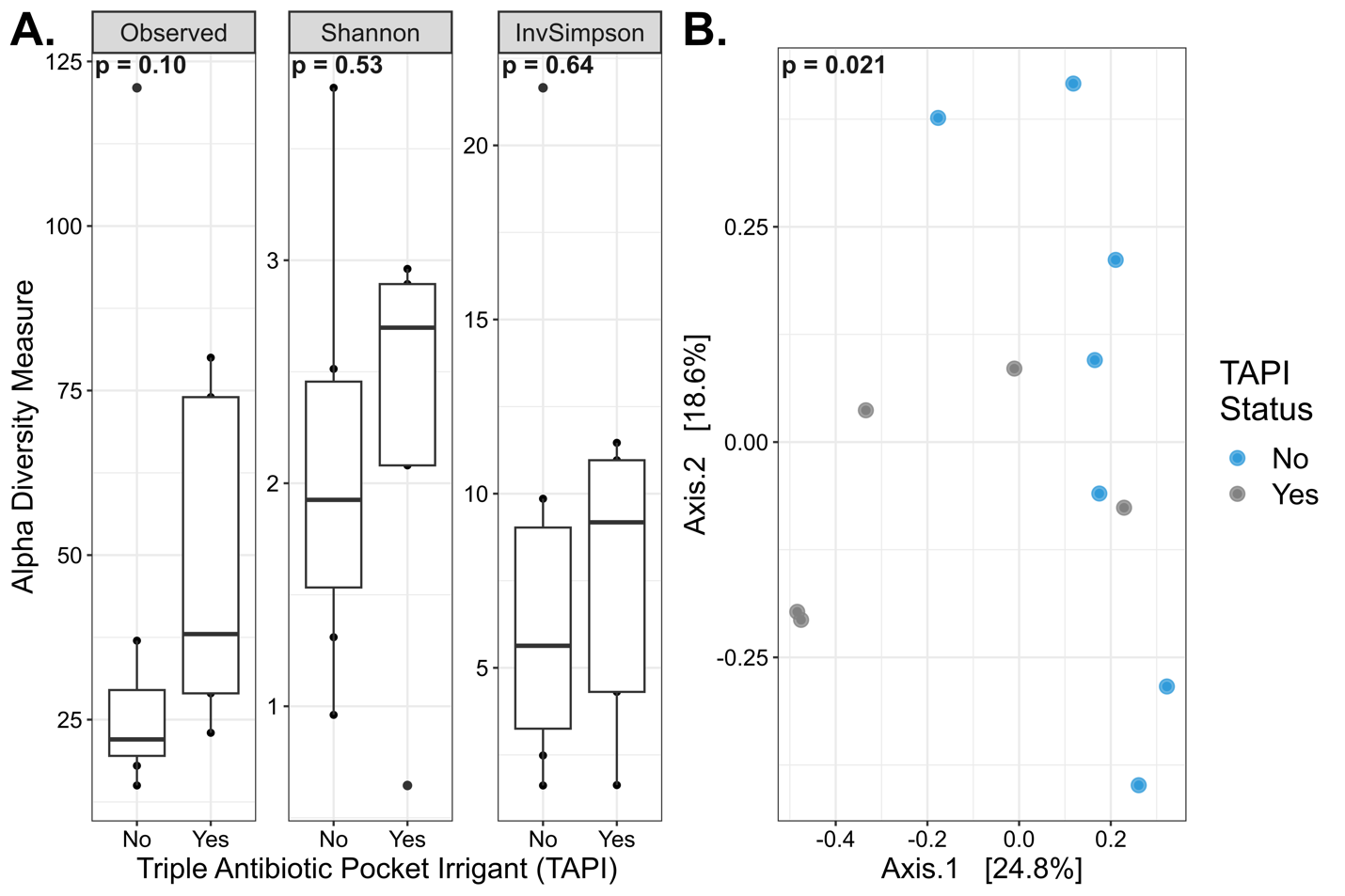
**

**Figure S1.** Alpha and beta diversity estimates were assessed for capsule samples from women without cancer, stratified by whether they received the triple antibiotic irrigant (TAPI) or the saline control. (A) For capsules from women without cancer a statistically significant difference was not observed for Observed, Shannon, and Inverse Simpson diversity between those who received TAPI and those who didn’t. Although, a consistent directionality of the association of lower alpha diversity in those who did not receive TAPI was observed across all three. (B) A principal component analysis demonstrates statistically significant differences in the clustering and centroids when comparing Beta Diversity of capsule samples by antibiotic status. Additionally, 43.4% of variability was accounted for on axes 1 and 2. The Wilcoxon-rank sum test was used to determine statistical significances, with *=p<0.05.


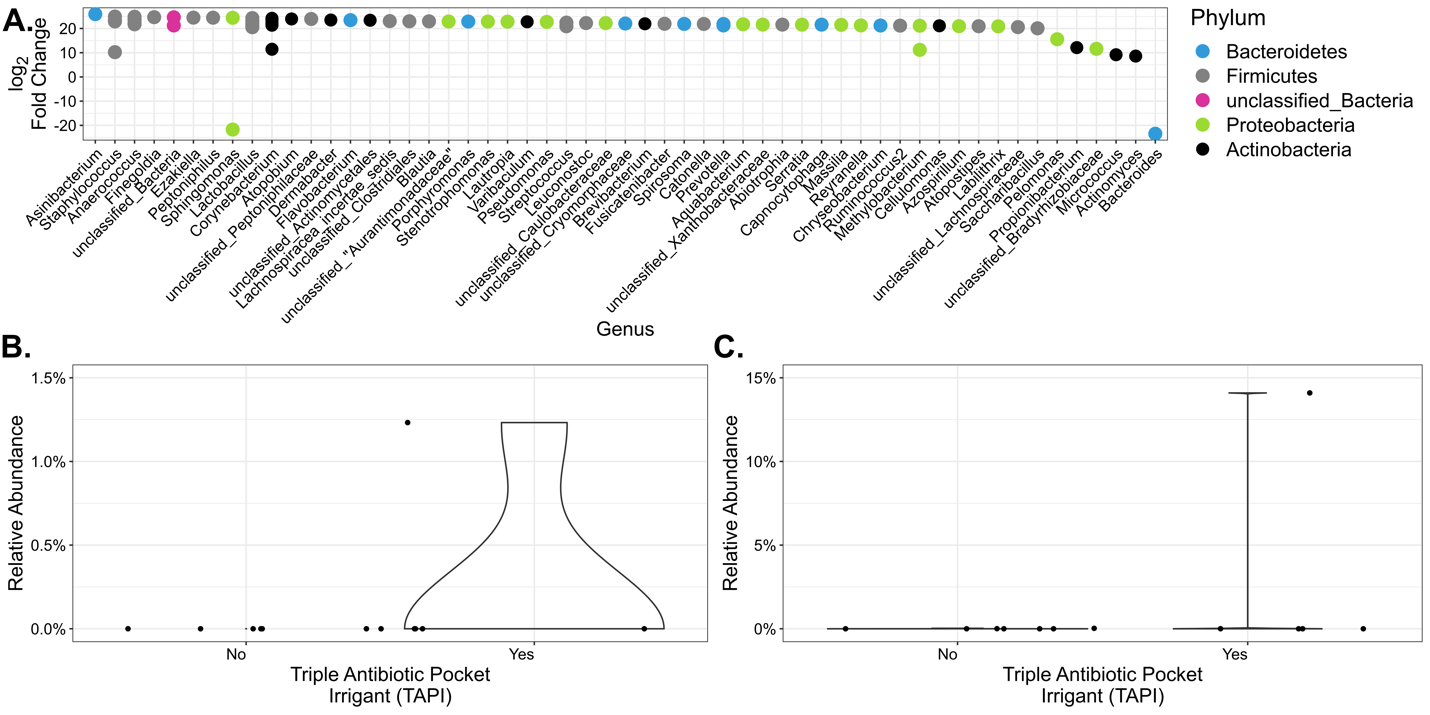


**Figure S2.** Differentially abundant taxa of capsules from women without cancer comparing those with and without TAPI identifies taxa whose prevalence (presence/absence) and relative abundance are very low. (A) The log2 fold change of taxa identified as differentially abundant. (B) Violin plots of the prevalence and the relative abundance of differentially abundant Staphylococcus OTU demonstrates the presence in one capsule from a woman who received TAPI, and none from women who received the saline control. (C) Violin plots of the prevalence and the relative abundance of differentially abundant *Cutiebacterium* OTU, with two capsules from women who received TAPI and two from women who received the saline control, with only one dominated by these taxa and the rest with relative abundances below five percent.


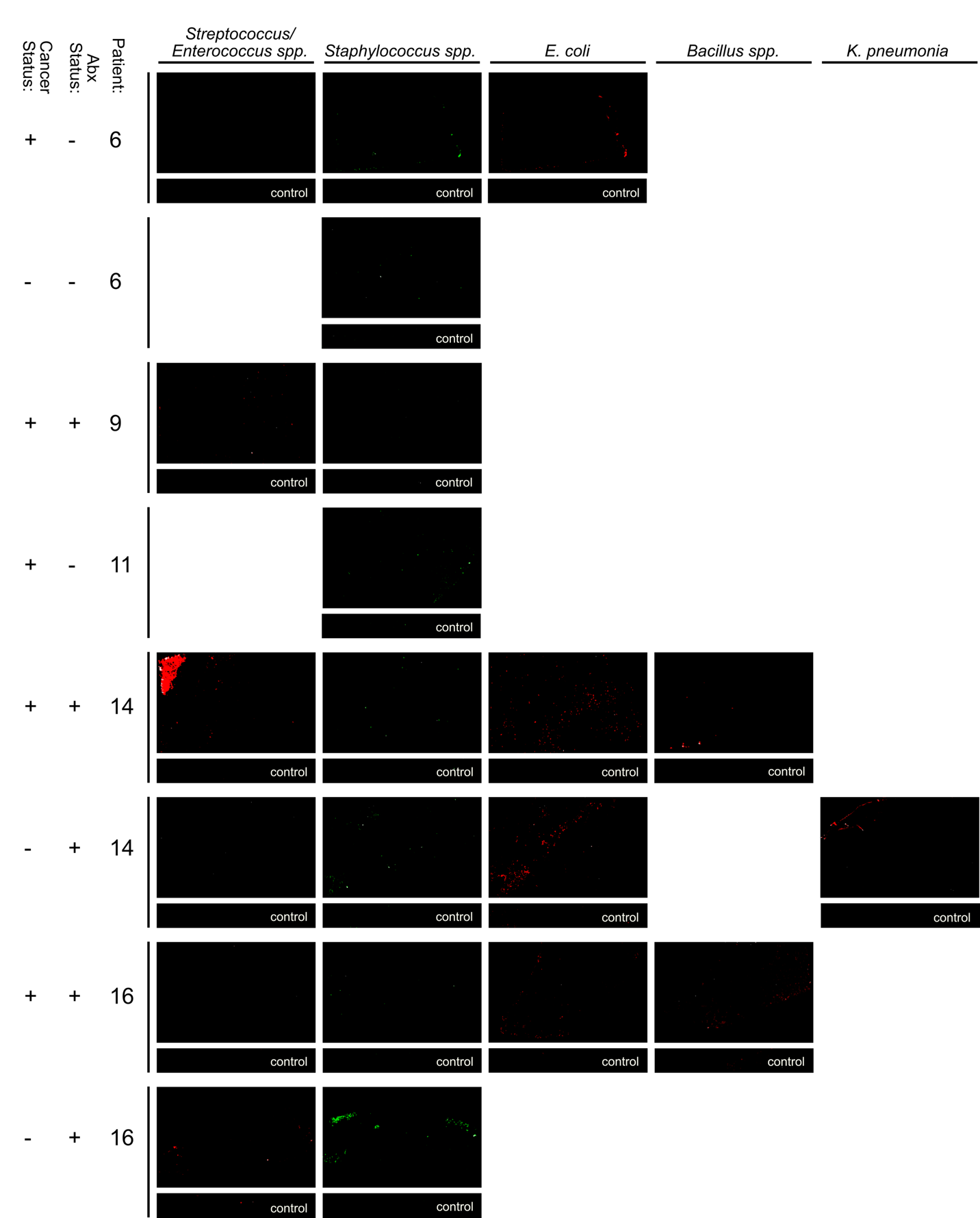


**Figure S3.** Representative immunofluorescence images of tissue expanders (TEs) stained for bacterial species identified via standard microbial culturing and/or 16S rRNA microbiome sequencing. Bacterial species, including staphylococci, enterococci, streptococci, and bacilli spp., as well as *E. coli* and *K. pneumonia,* could be visualized on TE surfaces.
